# Pulmonary Cryptococcus infections as a manifestation of idiopathic CD4 lymphocytopenia: case report and literature review

**DOI:** 10.1186/s12879-019-4453-x

**Published:** 2019-10-17

**Authors:** Christina S. Thornton, Oscar Larios, Jennifer Grossman, Thomas P. Griener, Steven Vaughan

**Affiliations:** 1 Division of Respirology, Department of Medicine, University of Calgary, 3330 Hospital Drive NW, Calgary, AB T2N 4N1 Canada; 2 Division of Infectious Diseases, Department of Medicine, University of Calgary, Calgary, AB Canada; 3 Division of Hematology and Hematological Malignancies, Department of Medicine, University of Calgary, Calgary, Alberta Canada; 4 Department of Pathology & Laboratory Medicine, Cumming School of Medicine, Department of Medicine, University of Calgary, Calgary, Alberta Canada

**Keywords:** Pulmonary cryptococcus, CD4 lymphocytopenia

## Abstract

**Background:**

Idiopathic CD4 lymphocytopenia (ICL) is a rare clinical disease with relative CD4 deficiency in the absence of HIV infection. The pathogenicity of ICL is poorly understood with an unclear incidence rate in the general population. Sequelae of ICL includes AIDS-defining infections, which most commonly includes *Cryptococcus neoformans.* Typically, *C. neoformans* infections present with CNS involvement but rarely with extra-CNS manifestations. Here, we present a rare case of ICL with exclusively primary pulmonary cryptococcus and a review of the literature.

**Case presentation:**

A 56-year-old female presented to our tertiary care hospital requiring a right hip open reduction intervention. The patient became febrile during admission, prompting a work-up that included a chest X-ray showing a peripheral pulmonary solitary nodule. Transthoracic biopsy revealed encapsulated yeast forms in keeping with *C. neoformans.* CD4 counts, repeated at least one month apart, were < 200 cells/mm^3^, with negative HIV testing. Flow cytometry and genetic testing were completed to elucidate the etiology of the immune deficiency, both of which were unremarkable. She was subsequently treated with 12 months of posaconazole with clinical resolution.

**Conclusions:**

Our patient highlights a rare clinical disease, which a review of literature revealed only five cases in the literature with exclusive pulmonary *Cryptococcus* in ICL/ This case demonstrates the strong clinical acumen required to properly diagnose and ultimately manage the patient.

## Background

Idiopathic CD4 lymphocytopenia (ICL) is a rare clinical disease with relative deficiency of CD4 T-cells in the absence of human immunodeficiency virus (HIV) 1 and 2 infections [1]. It is defined by the US Centers for Disease Control and Prevention (CDC) as a documented absolute CD4 T-lymphocyte count of < 300 cells/mm^3^ or < 20% of total T-cells on two separate time points at least six weeks apart [2]. Often, ICL is diagnosed in patients with opportunistic infections who test HIV negative.

*Cryptococcus neoformans* infection is the most common opportunistic infection [1] in ICL patients, however typically presents with central nervous system (CNS) manifestations. Here, we present a case of isolated pulmonary cryptococcus infection in a patient with ICL, along with an accompanying review of the literature. We highlight treatment modalities and diagnostic considerations for the clinician when faced with this rare disease.

## Case presentation

A 56-year-old female was admitted to a tertiary care hospital in Calgary, Alberta, Canada for a complex right total hip replacement after a mechanical fall. Her past medical history was significant for insulin dependent type 1 diabetes and a seizure disorder with remote left temporal lobectomy. She was a lifelong non-smoker with an otherwise unremarkable family, social and personal history. Travel history included visits to Vancouver Island, British Columbia.

Following operative intervention, on post-operative day 3, she was incidentally noted to be febrile at 38.4 °C and underwent routine investigations. Physical examination showed a healthy individual with unremarkable vital signs, including oxygen saturation on room air of 95%. Chest auscultation was clear. Neurological examination was within normal limits. Laboratory findings included hemoglobin count of 90 g/L, platelet count of 129 × 10^9^/L and white blood cell count 8.4 × 10^9^/L.

**Fig. 1:**
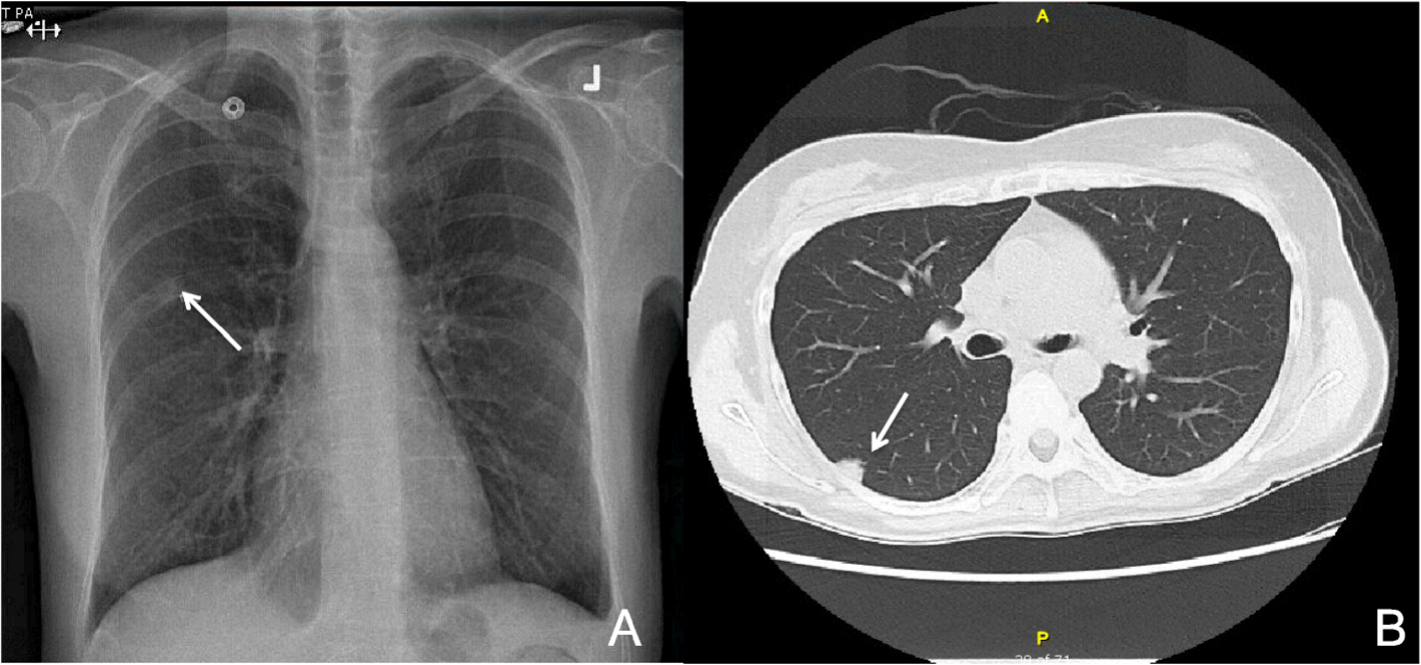
Chest radiograph demonstrating nodular opacification in the right upper lung (**a**). Chest computed tomography [3] scan demonstrating non-calcified and non-cavitating pleural-based lesion laterally in the superior segment of the right lower lobe measuring 14 × 11 mm (**b**)

A routine chest radiograph showed a new nodular opacification in the right upper lung that was not present in prior radiographs one year prior (Fig. 1a). Chest computed tomography [3] showed a small non-calcified and non-cavitating pleural-based lesion laterally in the superior segment of the right lower lobe measuring 14 × 11 mm (Fig. 1b). Margins were noted to be irregular with solid components. There was no axillary, mediastinal or hilar adenopathy identified. Given the new occurrence of this lesion, the radiology opinion favored peripheral small adenocarcinoma.

**Fig. 2:**
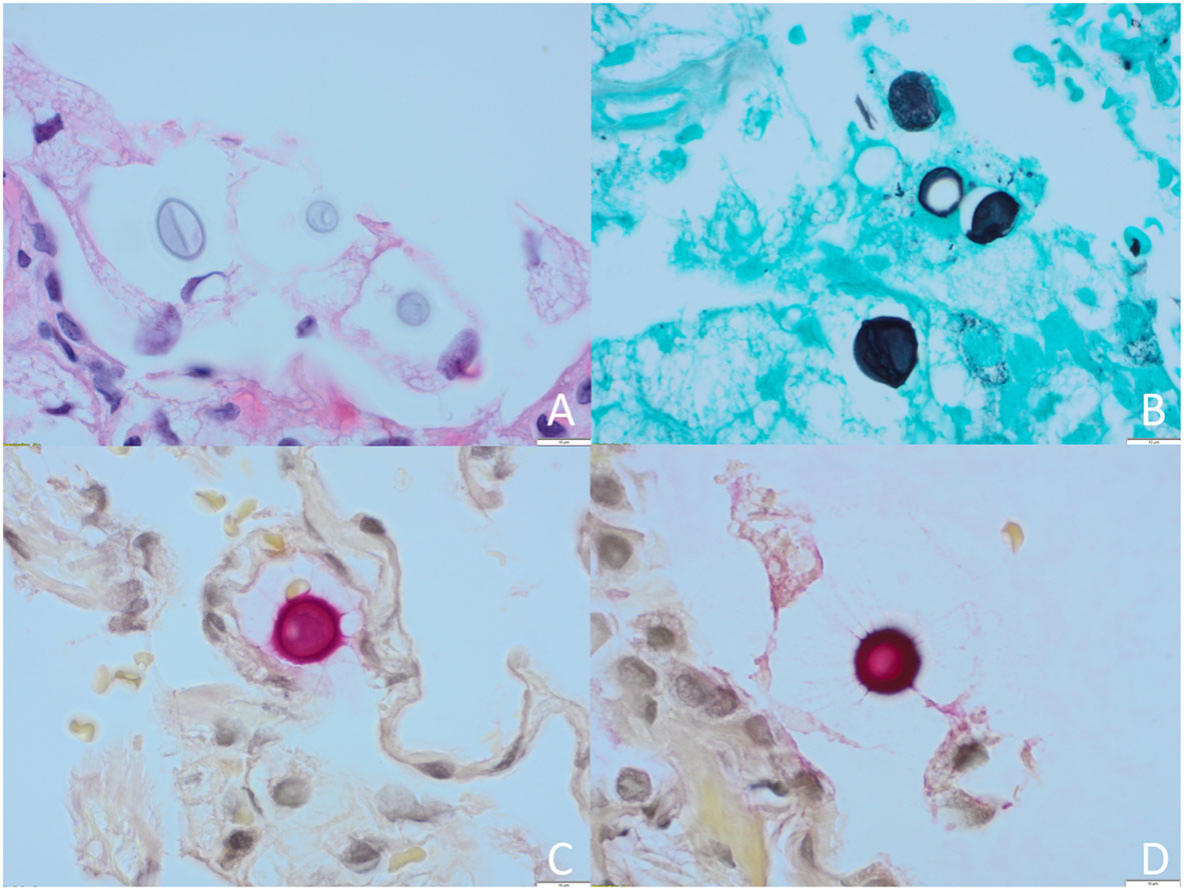
Pathologic examination of haematoxylin and eosin (H&E) stained lung tissue, revealing encapsulated and variably-sized oval yeast forms in a background of foamy histiocytes (**a**, 40x). Yeast morphology consistent with *Cryptococcus* spp. as the yeast cell wall stained positive by Grocott’s methenamine silver (GMS) stain (**b**, 50x) and large capsule positive by mucicarmine stain (**c**, **d**, 100x)

The patient underwent an interventional radiology guided transthoracic biopsy, which was complicated with development of small apical pneumothorax and subsequent chest tube insertion. Histological examination revealed multiple round-oval yeast forms admixed in a background of foamy macrophages. Budding yeast forms were not observed. As is typical for *Cryptococcus* spp., the yeast forms were variably-sized (ranging from 3 to 9 μm), the yeast cell wall stained positive by Grocott’s methenamine silver (GMS) and the large mucinous capsule was highlighted by mucicarmine staining (Fig. 2a-d). Fontana-Masson stain, which can be used to confirm the diagnosis by demonstrating the melanin pigmentation of *Cryptococcus,* was not performed.

The patient was subsequently seen in infectious diseases clinic. A serum cryptococcal antigen test was positive, with semi-quantitative titre of 1:40 (Cryptococcal Antigen Lateral Flow Assay, IMMY, Norman, Oklahoma). This assay is reported to detect both *C. neoformans* and *C. gattii* and has a high sensitivity in immunocompetent and immunocompromised individuals [4]. Further molecular sequencing was not available on the sample. She was started on fluconazole 400 mg oral daily. After initiation, she developed a severe headache and underwent lumbar puncture to rule out unmasking immune reconstitution syndrome from CNS cryptococcosis. Cerebrospinal fluid was negative for cryptococcal antigen and fungal culture. Due to the persistence of headache and a drug interaction with an anti-epileptic medication (carbamazepine) she was subsequently changed to posaconazole 300 mg oral daily. Cryptococcal antigen titres were followed in the year following initiation of posaconazole and decreased to negative levels at the end of treatment.

At time of diagnosis, a CD4 count was noted to be low at 0.286 × 10^^9^/L (0.499–1.651 × 10^^9^/L) with repeat four months later at 0.334 × 10^^9^/L. HIV testing with 4th Gen ELISA and HIV viral load were negative. An immunologic testing panel done 18 months after initial diagnosis showed normal total numbers of B-cells. Her total T-cell number was decreased at 0.33 × 10^^9^/L (0.780–3.00 × 10^^9^/L) with a CD4 count of 0.24 × 10^^9^/L (0.500–2.00 × 10^^9^/L) and CD8 count of 0.07 × 10^^9^/L (0.200–1.200 × 10^^9^/L). Neutrophil and NK cell counts were within normal limits. This was felt to be supportive of the diagnosis of idiopathic CD4 lymphocytopenia. DNA was tested for over 274 genes known to be associated with immune defects (Blueprint Genetics) and no mutations were found.

The patient ultimately completed 12 months of posaconazole monotherapy with excellent response. Repeat CT chest imaging done following completion of therapy showed the peripheral predominant ground-glass nodule within the superior segment of the right lower lobe as smaller and less dense when compared to prior measuring 12 × 8 compared to 14 × 11 mm. No further recurrence of *Cryptococcus* has occurred to date.

## Discussion and Conclusions

ICL is an exceedingly rare disease with unknown incidence in the general population. A review found that CD4 counts of ICL patients on initial diagnosis were < 150 cells/mm^3^ [5]. In contrast, AIDS is defined as an outcome of chronic HIV infection with CD4 count < 200 cells/μL or the presence of AIDS-defining conditions regardless of CD4 count. These include infections such as disseminated or extrapulmonary coccidioidomycosis, extrapulmonary cryptococcosis, disseminated mycobacterium and *Pneumocystis jiroveci* pneumonia [6]. In a review of 23,179 cases within the CDC immunodeficiency registry, 47 met criteria for ICL [7]. There was no reportable association with age (mean age 43 years, range from 17 to 78 years) or gender (male to female ratio of 29:18) amongst ICL patients. Currently, the etiology of ICL is unknown but has been speculated to range from a pathogenic process resulting in subsequent CD4+ apoptosis [8], T-cell kinase dysregulation [9] and mutations in the RAG GTPase pathway [10]. An unidentified genetic mechanism has also been suggested as there have been several reports with familial association [11, 12].

Opportunistic pathogens observed in ICL are similar to those in AIDS, however with differing frequencies. A review of 258 ICL patients found the most common to be *Cryptococcus* (26.6%), followed by mycobacterial (17.0%) and PJP (7.7%) [5]. In comparison, AIDS patients are most frequently infected with cytomegalovirus (33%) and PJP (29.9%), with only 2.6% cryptococcal infections [13, 14]. From a clinical perspective, the differences between the two diseases are relevant as ICL patients tend to have a progressive decline in CD4 counts, whereas AIDS patients exhibit a slower decline (or increase if antiretroviral medication is initiated) [8, 15].

As mentioned, *Cryptococcus neoformans* is the most common opportunistic infection amongst ICL patients. There have been reports of cryptococcal osteomyelitis, dermatological manifestation and musculoskeletal involvement [16–19]. Despite this, the vast majority of ICL patients with cryptococcal infections present with primarily CNS involvement. In a prospective American series of 39 patients, one-third of patients were diagnosed with cryptococcal meningitis [8]. A French group also found similar results in 15% of ICL patients [20]. While less common, reports of cryptococcal encephalitis have also been described [21].

Isolated pulmonary cryptococcosis without CNS involvement is rare with only, to our knowledge using a PRISMA review checklist, five cases in the literature reported (Table 1). Notably, treatment regiments were diverse within the cases. There is currently no gold standard for treatment of pulmonary *Cryptococcus* in ICL. Often, fluconazole is adequate treatment but the duration of therapy is unclear and should be tailored to the individual patient with severity of illness as guidance [22]. Guidelines for non-meningeal cryptococcal pulmonary infections published by the Infectious Diseases Society of America (IDSA) recommend for mild to moderate disease, administration of fluconazole 400 mg daily for 6–12 months with escalation to CNS therapy for severe disease [3]. Notably, persistently positive serum cryptococcal antigen titers are not criteria for continuous use of therapy. Given the limited cases available for pulmonary *Cryptococcus* in ICL, we suggest adoption of the IDSA guidelines as a framework for the clinician in treatment of these patients, as was done in our case.

**Table 1:** Summary of Literature Cases with ICL and Primary Pulmonary Manifestations

Reference	Year	Gender	Age	Clinical History	Treatment	Outcome
Ahn, et al [22].	2005	Male	73	Non-small cell lung cancer	Amphotericin B (0.5 mg/kg/day) for 15 days then switched to fluconazole 400 mg/day	Continued on fluconazole until lung nodules disappeared
Lin, et al [23].	1994	Female	33	Pure red cell aplasia and human parvovirus B19 infection	Brief course of amphotericin B followed by oral fluconazole.	Recurrent anemia requiring intravenous immunoglobulin for refractory anemia
McNulty, et al [24].	1991	Male	35	N/A	N/A	N/A
Zaharatos, et al [25].	2001	Male	47	Coinfection with *Mycobacterium tuberculosis*	N/A	Comment on 24 weeks total of therapy
Yuanjie, et al [26].	2008	Female	41	N/A	Lobectomy; amphotericin B 25 mg /day for 1 month; amphotericin B 25 mg/day plus 5-flucytosine 3 g /day for 6 weeks; amphotericin B 25 mg/day plus 5-flucytosine 3 g/day for 6 weeks; amphotericin B 25 mg/day plus 5-flucytosine 3 g/day for 12 weeks and fluconazole 150 mg/day as maintenance	Total of four relapses, maintained on oral fluconazole.

It is noted in the IDSA guidelines that in non-immunocompromised patients with pulmonary cryptococcosis, to consider a lumbar puncture to rule out asymptomatic CNS involvement. However, for normal hosts with asymptomatic pulmonary nodule or infiltrate, no CNS symptoms, and negative or very low serum cryptococcal antigen, a lumbar puncture can be avoided (B-II). It is unclear where patients with ICL fall into this spectrum. Given the high prevalence of CNS involvement in ICL patients, we would suggest the clinician strongly consider a lumbar puncture to rule out asymptomatic disease in all patients with extra-CNS cryptococcal infection.

Limitations of this study include the lack of sequencing or other molecular tests, highlighting an area of future work that may improve diagnostic utility in this rare disease.

## Data Availability

Not applicable.

## Abbreviations

AIDS: Acquired immunodeficiency syndrome
CD4: Cluster of differentiation 4
CNS: Central nervous system
CT: Chest computed tomography
GMS: Grocott’s methenamine silver
H&E: haematoxylin and eosin stain
HIV: Human immunodeficiency virus
ICL: Idiopathic CD4 lymphocytopenia
IDSA: Infectious Diseases Society of America
OI: Opportunistic infection
PJP: Pneumocystis jiroveci pneumonia
